# The Dose Rate of Corpuscular Ionizing Radiation Strongly Influences the Severity of DNA Damage, Cell Cycle Progression and Cellular Senescence in Human Epidermoid Carcinoma Cells

**DOI:** 10.3390/cimb46120828

**Published:** 2024-12-06

**Authors:** Sergey S. Soroko, Dmitry V. Skamnitskiy, Ekaterina N. Gorshkova, Olga M. Kutova, Ismail R. Seriev, Anna V. Maslennikova, Evgeniy L. Guryev, Sergey V. Gudkov, Vladimir A. Vodeneev, Irina V. Balalaeva, Natalia Yu Shilyagina

**Affiliations:** 1 Department of Biophysics, Institute of Biology and Biomedicine, Lobachevsky State University of Nizhny Novgorod, 23 Gagarin Ave., 603022 Nizhny Novgorod, Russia; soroko@unn.ru (S.S.S.); e.n.gorshkova@gmail.com (E.N.G.); kutovaom@gmail.com (O.M.K.); sirnnr@gmail.com (I.R.S.); s_makariy@rambler.ru (S.V.G.); v.vodeneev@mail.ru (V.A.V.); irin-b@mail.ru (I.V.B.); 2 Nizhniy Novgorod Regional Oncology Hospital, St. Rodionova, 190, 603950 Nizhny Novgorod, Russia; 3 Department of Oncology, Radiation Therapy and Radiation Diagnostics, Privolzhsky Research Medical University, Minin and Pozharsky Sq., 10/1, 603950 Nizhny Novgorod, Russia; 4 Prokhorov General Physics Institute of the Russian Academy of Sciences, Vavilov Str. 38, 119991 Moscow, Russia; 5 Federal Scientific Agronomic and Engineering Center VIM, 1st Institutsky Proezd 5, 109428 Moscow, Russia

**Keywords:** ionizing radiation, radiotherapy, cancer, low dose rate, high dose rate, mechanism cell response, beta-radiation, cell cycle, cell death, apoptosis, ROS, senescence, giant cells

## Abstract

Modern radiotherapy utilizes a broad range of sources of ionizing radiation, both low-dose-rate (LDR) and high-dose-rate (HDR). However, the mechanisms underlying specific dose-rate effects remain unclear, especially for corpuscular radiation. To address this issue, we have irradiated human epidermoid carcinoma A431 cells under LDR and HDR regimes. Reducing the dose rate has lower lethality at equal doses with HDR irradiation. The half-lethal dose after HDR irradiation was three times less than after LDR irradiation. The study of mechanisms showed that under HDR irradiation, the radiation-induced halt of mitosis with the accompanying emergence of giant cells was recorded. No such changes were recorded after LDR irradiation. The level of DNA damage is significantly greater after HDR irradiation, which may be the main reason for the different mechanisms of action of HDR and LDR irradiations. Comparing the mechanisms of cell response to LDR and HDR irradiations may shed light on the mechanisms of tumor cell response to ionizing radiation and answer the question of whether different dose rates within the same dose range can cause different clinical effects.

## 1. Introduction

Radiation therapy is administered to more than half of cancer patients as either an independent or auxiliary treatment option, with varying degrees of impact from radical to palliative [1,2,3]. To fit varying tasks and tumor localizations, different sources of ionizing radiation are chosen, such as classic photon and corpuscular radiation sources, sources possessing rare or dense ionizing radiation, and sources with different dose rates [4]. In the case of cutaneous malignancies, both external (total skin radiotherapy) and contact irradiation (brachytherapy) are applicable [5,6]. Sources for brachytherapy include gamma (^137^Cs and ^125^I) and beta (^90^Sr, ^90^Y, ^106^Ru, and ^60^Co) emitters of varying dose rates [7,8,9,10]. To date, the application of high-dose-rate (HDR) sources (300–2400 Gy/h) is common in brachytherapy [11,12,13]. At the same time, low-dose-rate (LDR) sources (less than 5 Gy/h) are also used quite often as continuous long-term irradiation, or in “hyperfractions” when irradiation is performed several times a day, and this approach is gaining popularity [14,15,16,17,18]. The effect of ionizing radiation on cells is mainly determined by the superposition of the direct and indirect (free radical-induced) damage of DNA and DNA repair mechanisms [19], and depends significantly on the particle fluence per unit time, which leads to the occurrence of dose rate effects [20]. Compared to HDR irradiation, LDR irradiation reduces the preponderance of DNA damage along the particle track in competition with repair processes, which should theoretically lead to less pronounced cell lethality. However, a number of reports provide controversial data, indicating “inverse dose-rate effects (IDREs)”, i.e., increasing cell death under LDR irradiation [21,22,23,24]. It is assumed that this might be due to the absence of a halt in cell cycle phases and cell–cell interactions [25,26,27]. On the other hand, massive DNA damage, induced under HDR irradiation, triggers the halt of mitosis, providing time for the cell to repair before division, and such recovery attempts lead to greater radiosensitivity [27]. There are a number of research articles confirming the difference in cell survival after HDR and LDR irradiations. When irradiated with a higher dose rate, a lower dose is required to achieve the effect. However, the mechanisms of the dose rate effect have not yet been studied. Such multifaceted theoretical causality of the irradiation outcomes under HDR and LDR irradiations, and the actual scarcity of information about the distinction between the underlying mechanisms of each, especially for corpuscular radiation, inspires new research endeavors.

The purpose of our study is to compare the mechanisms of cell response to LDR and HDR corpuscular irradiations. From a radiobiology perspective, comparisons of the biological effects of exposure to HDR and LDR irradiations will allow us to gain a more complete understanding of the cellular and molecular mechanisms of radiation damage. In turn, the acquired knowledge will need to be taken into account when transferring technologies into clinical practice.

## 2. Materials and Methods

### 2.1. Cell Culture

For this study, we used the human epidermoid carcinoma A431 cell line (All-Russian Collection of Cell Cultures, Institute of Cytology of the Russian Academy of Sciences, Saint-Petersburg, Russia). This cell type is a suitable model because radiotherapy has been widely used for the treatment of tumors of epidermal origin [28]. It is worth noting that A431 is a skin squamous cell carcinoma. This type of cancer is treated in combination with radiotherapy, and brachytherapy methods are also used for treatment [29,30,31,32,33,34,35].

The cells were cultured in 25 cm^2^ culture flasks (Corning, Corning, NY, USA) in DMEM (HyClone, Logan, UT, USA) with 10% fetal bovine serum (HyClone, USA) and 2 mM L-glutamine (PanEco, Moscow, Russia) at 37 °C and 5% CO_2_. For passaging, the cells were removed from the culture flask using a trypsin-EDTA solution (1:1) (PanEco, Russia), and washed with 10 mM PBS. The cells were passaged when the culture reached 80% confluence.

### 2.2. Radiation Sources and Irradiation Regimes

The HDR corpuscular irradiation of A431 human epidermoid carcinoma cells seeded into culture plates was performed in Nizhniy Novgorod Regional Oncology Hospital using a linear accelerator Novalis Tx (Varian, Palo Alto, CA, USA) with an electron energy of 6 MeV. The source-to-surface distance (SSD) was 100 cm, the field size was 25 × 25 cm^2^, and the dose rate was 600 Gy/h. The radiation dose was controlled by the exposure time, which did not exceed 10 min. The calculation of the delivered dose was performed using the Electron Monte Carlo eMC algorithm. Control cells and irradiated cells were seeded to separate plates, so the control cells were not irradiated.

**Figure 1:**
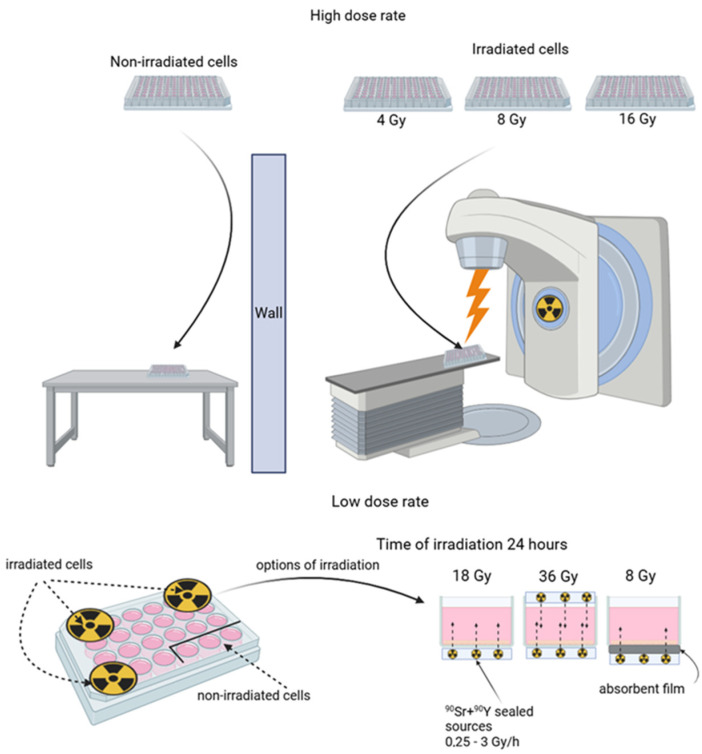
Scheme of the irradiation of A431 human epidermoid carcinoma cells. High-dose-rate irradiation (**top panel**) was performed using linear accelerator Novalis Tx (Varian, USA); different doses were achieved under fixed device settings by varying exposure time, which did not exceed 10 min. Low-dose-rate irradiation (**bottom panel**) was performed using beta-emitting ^90^Sr + ^90^Y sealed sources; dose precision was achieved under a fixed exposure time of 24 h by the combined use of sources of different activities. The control cells were protected by liquid shielding. For further details, please see Section 2.

The LDR irradiation of A431 human epidermoid carcinoma cells was performed using beta-emitting ^90^Sr + ^90^Y sealed sources. The half-life of the ^90^Sr isotope is 28.8 years, and the maximum decay energy is 0.5 MeV. The half-life of the ^90^Y isotope is 64 h, and the maximum decay energy is 2.3 MeV [36]. We used the sealed sources with the activity of 1 MBq on a polypropylene surface with a working zone of Ø 10 mm and 3 MBq on a steel surface with a working zone of Ø 24 mm (FSUE PA Mayak, Ozyorsk, Russia). The dose was controlled by the power of the source (a combination of several sources above and/or below the well and shielding with films). The radiation dose rate varied from 0.25 to 3 Gy/h. The irradiation time remained constant and was 24 h (Table 1). During irradiation, cell culture confluence did not exceed 50%. In the experiments with LDR irradiation, both the control and irradiated cells were located on one culture plate. To ensure adequate protection of the control cells from irradiation, the control cells were seeded to the farthest wells of the plate from the wells containing the cells that were to be irradiated. The rest of the wells were filled with water, thus making a liquid shield (Figure 1).

### 2.3. MTT Assay

**Figure 2:**
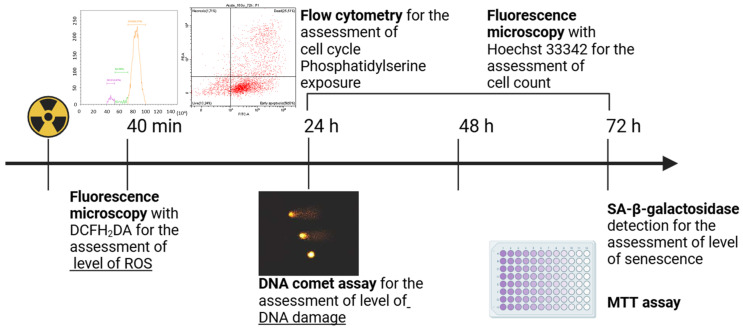
Assessment of the vital functions of the irradiated A431 human epidermoid carcinoma cells. The level of reactive oxygen species (ROS) was assessed 40 min after the start of irradiation by fluorescence microscopy using the ROS indicator DCFH_2_DA; the level of DNA damage was assessed 24 h after the start of irradiation by the DNA comet assay; cell cycle and cell death type were analyzed 24, 48, and 72 h after the start of irradiation by flow cytometry; the number of cells in the culture was counted 24, 48, and 72 h after the start of irradiation using Hoechst 33342 by fluorescence microscopy; cell count was assessed 72 h after the start of irradiation by the MTT assay; the degree of cellular senescence was assessed 72 h after the start of irradiation by the enzymatic staining of cells for SA-β-galactosidase. For further details, please see Section 2.

The A431 cells were seeded in the 96-well culture plates at a concentration of 500 cells per well in DMEM with 10% FBS and were let to attach overnight. Then, irradiation was performed at a dose of 2, 4, 8, 16, 32, 36, and 64 Gy for the HDR and 2, 4, 8, 12, 24, and 32 Gy for the LDR regimes, respectively. A duration of 72 h after the start of irradiation, the count of living cells was estimated using the MTT assay. The medium was replaced with the fresh serum-free medium containing 0.5 mg/mL MTT (Alfa Aesar, Ward Hill, MA, USA) followed by incubation for 3 h. Formazan formed from the reduction of MTT by mitochondrial dehydrogenases was dissolved in dimethyl sulfoxide (PanEco, Russia). The optical density of the resulting solutions at a wavelength of 570 nm was measured using a Synergy MX plate reader (BioTek, Shoreline, WA, USA). The count of living cells was calculated as the percentage of mean optical density in the wells with the irradiated cells to the mean optical density in the wells with the non-irradiated cells. Dose–response curves were fitted by nonlinear regression using a four-parameter dose–response model. The resulting dose–response ratios were used to calculate the LD_50_ (the radiation dose permitting 50% living cells) and D_37_ (the radiation dose permitting 37% living cells) using a four-parameter lognormal distribution model. All the calculations were performed using the GraphPad Prism 9 software. The values of LD_50_ and D_37_ were used as a reference for the choice of irradiation doses for further experiments (Figure 2). The MTT assay does not assess cell death. The results of the MTT assay can be affected by delays in cell division and changes in their metabolic status [37,38]. To assess viability in the case of radiation exposure, the MTT assay can give false results [39], and therefore, we conducted additional cell viability tests.

### 2.4. Fluorescent Microscopy for Number of Cells in Culture

The number of cells in the culture was assessed with the vital fluorescent dye Hoechst 33342. For the analysis, doses close to LD_50_ and D_37_ were chosen for each of the irradiation regimes. HDR irradiation was performed at doses of 4 Gy (LD_50_), 8 Gy (D_37_), and 16 Gy (>D_37_). LDR irradiation was performed at doses of 12 Gy (LD_50_) and 36 Gy (>D_37_). The analysis was carried out every day for 72 h. The cells were incubated with Hoechst 33342 (0.1 μg/mL; Thermofisher, Waltham, MA, USA) for 15 min. Hoechst 33342 fluorescence was visualized using the fluorescence microscope Axio Vert A1 (Carl Zeiss, Jena, Germany) (λex 365 nm, λem 445/50 nm); the resulting images were uploaded to the Image J software v. 1.54g (National Institutes of Health, Bethesda, MD, USA) and the stained cells were counted. The increase rate of the number of cells was estimated using the growth rate coefficient which was calculated as follows (Equation (1)):

(1) $$k=\frac{\ln\left(\alpha_{t}\right)-\ln\left(\alpha_{0}\right)}{t}$$

where α*_t_*—the number of cells after time t; α_0_—the initial number of cells; and t—the time after the start of irradiation.

### 2.5. Clonogenic Assay

For a clonogenic analysis, 6 h after exposure to ionizing radiation, the irradiated cells were seeded in culture dishes Ø 40 mm (Corning, USA) with a density of 10,000 cells per well. The cells were incubated for 7 days, renewing the culture medium every 3 days. Upon the completion of incubation, the cells were fixed in a 10% formaldehyde solution at room temperature for 20 min, after which they were stained with a 0.5% solution of crystal violet (LenReaktiv, St Petersburg, Russia). To remove excess dye, the colonies were washed with distilled water and left to dry completely at room temperature. The analysis of the area occupied by cell colonies in each well was carried out using the ImageJ software version 1.54f (National Institutes of Health, USA). The relative clonogenic activity was calculated as the ratio of the mean area of colonies in the experimental groups to the control group. GraphPad Prism version 9.0 (GraphPad Software, La Jolla, CA, USA) was used for the statistical analysis of the data.

When counting the total number of cells in culture and in the clonogenic analysis, we did not assess only cell death. Cytotoxic, cytostatic, and cytomodifying (such as giant cell formation or senescence) effects contribute to the results of the assays [37,40,41]. We could not assess the root cause of the decrease in the number of live cells in the sample and their condition by the methods used.

### 2.6. ROS Assay in Cells

The cells were seeded in 96-well culture plates at a concentration of 5 × 10^3^ cells per well in DMEM with 10% FBS. The catalase (0.1 mg/mL) was added 1 h before irradiation. A duration of 40 min after the end of irradiation, DCFH2DA (10 μM; Sigma-Aldrich, St. Louis, MO, USA) was added to the cells and incubated for 20 min. Images were obtained using the confocal laser scanning microscope Axio Observer Z1 LSM-710 DUO NLO (Carl Zeiss, Germany) (λ_ex_ 488 nm, λ_em_ 493–625 nm) and processed using the Zen Blue software v. 3.0 (Carl Zeiss, Jena, Germany). At least 10 cells were analyzed in each of the 10 fields of view.

### 2.7. Analysis of DNA Damage

To assess DNA damage in the irradiated cells, we used an alkaline version of the comet test with some modifications [28]. Briefly, in negatively charged DNA containing breaks, due to the relaxation of the supercoils, broken ends migrate toward the anode during electrophoresis. When tagged with a fluorescent dye, such damaged DNA is seen as a comet, where the tail contains leading ends of migrating fragments [29]. The cells were irradiated at doses of 4 and 8 Gy LDR and HDR. To perform the comet assay, the irradiated cells were embedded in low-melting agarose (LMA) gels. All the slides and coverslips used to obtain gels were coated with 1% universal agarose for optimal adhesion. After irradiation, 30 μL of cell suspension (≈0.1–1 × 10^5^ cells/mL) was mixed at 25 °C with an equal volume of 1% low-melting agarose (LMA) solution prepared in PbEDTA. For each sample, 3 microscopy slides were prepared. To prepare 3-layer slides, 15 μL of a mixture of cell suspension with 1% LMA was applied to each slide (under a coverslip), pre-coated with 0.5% LMA. After the agarose with cells solidified (5 min at 4 °C), a top layer of 0.5% LMA (15 μL) was applied and left for 5 min at 4 °C to solidify. The final layer was applied to fill any voids and to ensure that the cells were evenly distributed in the agarose after lysis. The uniform thickness of all 3 layers on the slides was achieved using special stands with a fixed depth of 50 µm. After the top layer of agarose solidified, cell lysis and DNA deproteinization were performed. The slides were placed in a lysis solution (2.5 M NaCl, 10 mM Tris-HCl, 1% Triton X-100, 1% N-lauryl-sarcosine, and 100 mM EDTA; pH = 10.0) for 25 min at 37 °C in the dark. Next, to separate the DNA strands and convert the existing alkali-labile sites into single-strand breaks, the preparations were kept in an alkaline solution (0.3 M NaOH and 1 mM EDTA; pH > 13.0) for 20 min at 4 °C in the dark. Electrophoresis was carried out in a fresh portion of an alkaline solution at 4 °C for 20 min at an electric field strength of 3.0 V/cm. The slides were then washed twice for 5 min in distilled water and stained with ethidium bromide (1 μg/mL in PBS) for 1 h at room temperature in the dark. Before analysis, each slide was washed for 5 min in distilled water and placed under a coverslip. The slides were examined using the fluorescent microscope MIKMED-2 version 11-1 (LOMO, Russia) (pass filter 525 nm, cut-off filter 590 nm). A camera with a sensitivity of 0.001 Lux was used to capture images of nucleoids. Image analysis was performed using the Comet software v1.3.1 with an image analysis speed of up to 400 comets/h. Automated solutions were used to analyze the number of DNA plumes (tails) obtained as a result of the DNA comet procedure. This made it possible to reduce the subjectivity of assessment and more accurately assess the size and shape of the formed plumes, which is especially important given the need to collect data on a large number of plumes in each sample (https://cometbio.org/, accessed on 28 November 2024). The degree of DNA damage was determined by the ratio of the plume to the total amount of DNA in percentage.

### 2.8. Analysis of Cellular Senescence

Cellular senescence was analyzed according to the degree of cell staining with the beta-galactosidase-processed substrate performed 72 h after the start of irradiation using a commercial Senescence β-Galactosidase Staining kit (Cell Signaling Technology, Danvers, MA, USA) according to the manufacturer’s recommendations. Micrographs of the cells were obtained using a wide-field microscope Axio Vert A1 (Carl Zeiss, Germany). The degree of cell staining was assessed using the Image J software v. 1.54g (LOCI, University of Wisconsin, Madison, WI, USA). The color intensity was calculated from the 8-bit image as a percentage relative to the maximum intensity value of 256.

### 2.9. Analysis of Phosphatidylserine Exposure on Cell Surface

Phosphatidylserine exposure on the cell surface was determined 24, 48, and 72 h after the start of irradiation by flow cytometry using the Annexin V apoptosis marker conjugated to fluorescein isothiocyanate (FITC) and the propidium iodide (PI) dye Annexin V Apoptosis Detection Kit I (BD Biosciences, Franklin Lakes, NJ, USA).

Based on the staining with PI and AnnexinV-FITC dyes, groups of cells were identified according to their state. “Living” cells were considered to be negatively stained (unstained) by both dyes—«PI-AnnV-». The cells positively stained with Annexin V were considered to be the cells with the presence of phosphatidylserine on the outer side of the membrane. Positively stained PI cells were considered to be the cells with a violation of the integrity of the cell membrane. Thus, four groups of cells were formed: «PI-AnnV-» labeled as «living» cells; «PI-AnnV+» labeled as «early apoptotic» cells; and «PI+» labeled as «dead» cells.

Due to the long-term cultivation of the cells post-irradiation, specific seeding densities were selected to maintain an 80% confluence of cell cultures on the day of the assessment of the cell death status for each irradiation regime [30]. The cells subjected to HDR irradiation were seeded in 6-well culture plates at concentrations of 10^5^, 5 × 10^4^, or 2 × 10^4^ cells per well; and the cells subjected to LDR irradiation were seeded in 24-well culture plates at concentrations of 4 × 10^4^, 2 × 10^4^, or 10^4^ cells per well and were let to attach overnight. Then, the irradiation was carried out at doses of 8, 16, and 32 for HDR irradiation and at doses of 12 and 36 Gy for LDR irradiation. Attached and free-floating cells were collected for analysis. The attached cells were detached using an EDTA solution. The cells were washed twice with cold PBS and centrifuged for 5 min at 200× *g*. The pellet was resuspended in Annexin V binding buffer at a final concentration of 1.5 × 10^5^ cells per 100 μL. A total of 100 μL of the resulting solution was transferred to a culture tube and 5 μL of Annexin V-FITC and propidium iodide (PI) were added. The cells were incubated for 15 min at 25 °C in the dark. A total of 400 μL of the tenfold diluted Annexin V binding buffer was added to each tube. The cells were analyzed within an hour using a CytoFLEX S flow cytometer (Beckman Coulter, Brea, CA, USA).

The fluorescence of FITC and PI was excited by a 488 nm laser, and the signal was detected in the range of 505–545 nm for FITC and in the range of 564–606 nm for PI. In total, 2 × 10^4^ events were analyzed, and debris and clumped cells were excluded from the analysis. The processing and analysis of the results was carried out using the CytExpert 2.3 software (Beckman Coulter, USA).

### 2.10. Cell Cycle Assay

The distribution of cells by the stages of the cell cycle was determined 24, 48, and 72 h after the start of irradiation using flow cytometry. Cell seeding and subsequent irradiation were performed similarly to the technique described in the previous section. Attached and free-floating cells were collected for analysis. The cells were washed twice with cold PBS and centrifuged for 5 min at 200× *g*. Staining was performed using the BD Pharmingen™ Cell Cycle Kit (BD Biosciences, USA) 24, 48, and 72 h after the start of irradiation according to the manufacturer’s protocol, and the cells were analyzed using a CytoFLEX S flow cytometer (Beckman Coulter, USA).

PI fluorescence was excited with a 488 nm laser and detected in the range of 564–606 nm. A total of 2 × 10^4^ events were analyzed; debris and clumped cells were excluded from the analysis. The processing and analysis of the results was carried out by the CytExpert 2.3 software (Beckman Coulter, USA). The level of PI fluorescence reflects the amount of DNA in the cell, which allows the pre-replication phase, the replication period, and the post-replication phase of DNA to be separated from each other.

### 2.11. Count of Giant Cells

The number of giant cells was assessed by fluorescent microscopy and flow cytometry. The investigation was made on an A431 cell line with a genetically encoded fluorescent protein GFP (λem = 475–550 nm, green) expressed in the cytoplasm (Figure S1). The protein was used to contrast and more accurately determine the boundaries and sizes of the cells. For each method, the sample preparation was described above. The cells exceeding the diameter of cells in the control group by 2 times (more than 30 μm) on fluorescence images were considered giant cells. Using flow cytometry, the giant cells were identified by high values of the fsc-a and fsc-h signal relative to the population of the non-irradiated cells.

### 2.12. Statistical Analysis

Statistical analysis was performed using the GraphPad Prism v.9.0 software (GraphPad Software, USA). Flow cytometry data were processed using the CytExpert 2.3 software (Beckman Coulter, USA). Statistical analysis is performed using the ANOVA Dunnett test to compare a group of unirradiated cells and those irradiated with different doses.

## 3. Results

### 3.1. LDR and HDR Effects on A431 Cells

Human epidermoid carcinoma A431 cells were irradiated under the LDR (0.5–3 Gy/h) regime and HDR (600 Gy/h) regime in the same dose range (2–64 Gy). The LDR and HDR effects on the A431 cells were assessed 72 h after the start of irradiation since irradiation-induced epidermoid cell death typically occurs after 2–3 division cycles [42]. We have previously shown that the doubling period of the A431 cells in culture is 26 h [43]. Thus, the time point of 72 h after the start of irradiation allowed us to evaluate the cell response after several culture doubling cycles [43].

**Figure 3:**
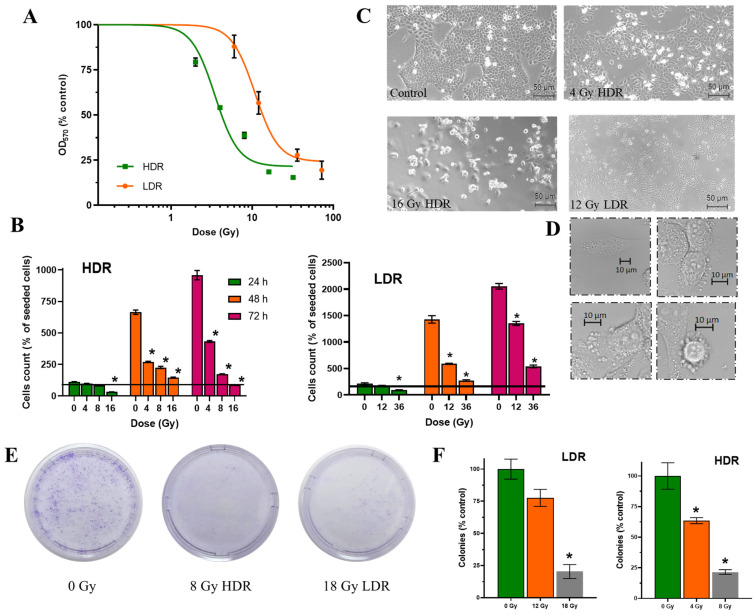
Effects of HDR/LDR irradiation on the A431 cells. (**A**)—dose–response curves 72 h after HDR and LDR irradiations (MTT assay); (**B**)—the percentage of live cells in culture 24, 48, and 72 h after each type of irradiation. The line indicates the number of initially attached cells in culture, right before irradiation (fluorescence microscopy). Data are represented as mean ± CV (*n* = 3 independent biological experiments), where “CV” is the coefficient of variation. “*” indicates a significant difference in cell viability between the non-irradiated and irradiated cells (ANOVA and Dunnett’s test, *p* < 0.0001); (**C**)—micrographs of the A431 cells 72 h after HDR and LDR irradiations at various doses; (**D**)—representative images of the typical morphological features of apoptosis in HDR-irradiated cells; (**E**)—representative images of culture dishes with stained cell colonies; (**F**)—the percentage of live cells in culture (clonogenic assay). “*” indicates a significant difference in colony area between the non-irradiated and irradiated cells (ANOVA and Dunnett’s test, *p* < 0.0001).

After irradiation in the LDR and HDR modes, a dose-dependent decrease in the number of cells was observed 72 h after the start of irradiation (Figure 3A). The analysis of the dose–response curves allowed us to calculate LD_50_ and D_37_ for both irradiation regimes. After LDR irradiation, LD_50_ reached ~10.8 Gy and D_37_ ~20 Gy, while after HDR irradiation, LD_50_ was 3.4 Gy and D_37_ ~8 Gy. I.e., after LDR irradiation, LD_50_ and D_37_ values were three times higher than after HDR irradiation. In the case of HDR irradiation, equal effects on the number of cells were observed at significantly lower doses compared to LDR. We assume that the response of cells to irradiation in different exposure modes develops according to different scenarios, which leads to differences in the ranges of cell radiosensitivity for each exposure mode.

### 3.2. Count of Living Cells

Due to the dependence of tetrazole stain-based tests on the metabolic status of cells which can lead to erroneously high levels of evaluated inhibitory effect, we have additionally stained attached cells with vital stain Hoechst 33342 and counted cells using fluorescence microscopy. For the analysis, doses close to LD_50_ and D_37_ were chosen for each of the irradiation regimes. HDR irradiation was performed at doses of 4 Gy (LD_50_ MTT assay), 8 Gy (D_37_ MTT assay), and 16 Gy (>D_37_ MTT assay). LDR irradiation was performed at doses of 12 Gy (LD_50_ MTT assay) and 36 Gy (>D_37_ MTT assay).

After irradiation, a dose-dependent decrease in the rate of cell growth in culture was observed; however, the dynamics of the decrease depended on the dose rate (Table 2, Figure 3B).

It is important to note that after irradiation at a dose >D_37_, a decrease in the number of cells in culture was observed already 24 h after irradiation both under the HDR and LDR regimes. A pronounced prolonged effect of ionizing radiation, namely a decrease in the number of cells in culture 72 h after irradiation, was observed only after HDR exposure. This decrease in cell number is clearly due to ongoing cell death in the culture rather than due to the combined effect of cell death and the halt of cell division. At ≥D37, the number of cells in the culture was halved between 48h and 72 h after irradiation.

The results of the two methods for assessing cell count together allowed us to evaluate both the general radiosensitivity of the cells in culture and the dynamics of culture growth after irradiation. In our opinion, it is important to take into account not only the fact of cell death after irradiation, but also the contribution of the delayed effects of radiation, which, apparently, prevail after HDR irradiation.

### 3.3. Number of Coloniesssa

We estimated the proliferative capacity of cell culture using the classical radiobiological method—clonogenic analysis. The data obtained are in very good agreement with previous analyses, although they differ slightly in values. We can see that the surviving fraction after irradiation at doses of 4 Gy for HDR and 12 Gy for LDR exceeds 50%, which is more than in the previous assays. At the same time, radiation exposure at doses of 8 Gy for HDR and 18 Gy for LDR is at the level of 25%, which corresponds to the values of the previous methods of analysis. It can be assumed that exposure at doses close to LD_50_ results in sufficient cell death, but does not lead to a significant loss of proliferative capacity, which allows cells to divide and form colonies after both modes of exposure (Figure 3E,F).

### 3.4. Morphological State of Cells

The non-irradiated cells retained typical morphology as they were attached to the substrate, flattened, and formed intercellular contacts. This morphology was maintained throughout the observation period, and with increasing confluence, the number of intercellular interactions increased. The irradiated cells exhibited signs of apoptotic death (detachment of the cells from the substrate, collapsing, swelling, or cellular blebbing) (Figure 3C,D).

### 3.5. Cell Cycle

**Figure 4:**
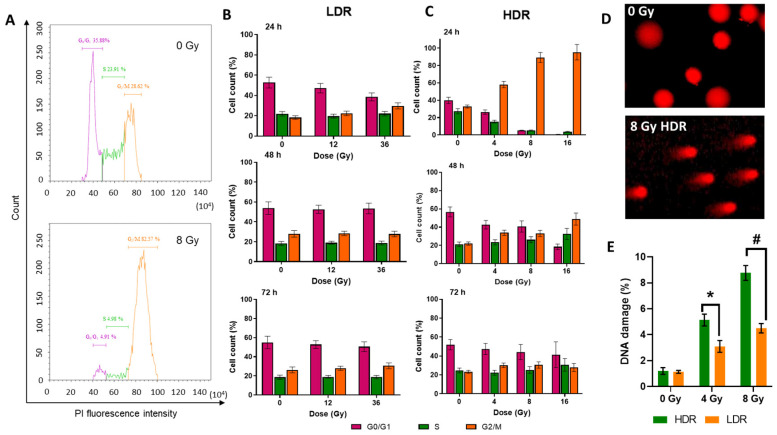
Flow cytometry evaluation of the cell cycle phases of the A431 cells analyzed by propidium iodide (PI) staining and DNA breaks analyzed by the DNA comet test: (**A**)—example of the distribution of cells by cell cycle phases depending on the amount of stained DNA in the non-irradiated cells, and cells under HDR irradiation after 24 h; (**B**)—distribution of the A431 cells by cell cycle phases 24, 48, and 72 h after LDR irradiation at various doses (**C**)—distribution of the A431 cells by cell cycle phases 24, 48, and 72 h after HDR irradiation in various doses. Data are represented as mean ± CV (n = 3 independent biological experiments), where “CV” is the coefficient of variation; (**D**)—representative images of comets for the DNA damage comet assay; (**E**)—the percentage of DNA breaks occurring after HDR and LDR irradiations after 24 h. Data are represented as mean ± CV (n = 3 independent biological experiments), where “CV” is the coefficient of variation. “*” and “#” indicate significant differences in the percentage of DNA damage between the non-irradiated cells and cells irradiated at doses of 4 Gy and 8 Gy, respectively (ANOVA and Dunnett’s test, *p* < 0.05; # *p* < 0.0001).

The non-irradiated cells were shown to fall into a typical distribution for untreated cell culture where half of the population is in the G0/G1 phase and the other half is roughly divided into quarters by S or G2/M phases. A similar picture was observed in the cell cultures subjected to LDR irradiation; thus, under LDR irradiation, the cell cycle was unaltered under either selected dose of irradiation (Figure 4B). On the contrary, the cells exposed to HDR irradiation underwent a dose-dependent arrest in the G2/M phase 24 h after irradiation (Figure 4A,C). At the dose of 4 Gy, the number of cells in the G2/M phase has increased by 25–50% of the total number of living cells, and at doses of 8 and 16 Gy, it reached almost 100%. The restoration of normal cell cycle phase distribution was observed 48 h after HDR irradiation at doses of 4 and 8 Gy, and 72 h after irradiation at a dose of 16 Gy.

Thus, the absence of a radiation-induced halt of mitosis after LDR irradiation and its presence after HDR irradiation were observed. The severity of the radiation-induced halt of mitosis after HDR irradiation depended on the radiation dose: a higher dose produced a greater effect. The duration of the radiation-induced halt of mitosis was also dose-dependent. For lower doses (<D_37_), the ratio of cells in cell cycle phases was restored after 48 h, and for higher doses, after 72 h.

It is also important to note that with an increase in the dose and time elapsed after HDR irradiation, the amount of cellular debris that did not fall into the analyzed gate also increased, which not only indicates the restoration of proliferative activity, but also the presence of delayed cell death as a result of irradiation.

In both irradiation modes, we observed the presence of DNA damage in the form of strand breaks. However, it is clearly seen that HDR irradiation causes twice as much DNA damage as LDR irradiation at the same radiation doses (Figure 4D). In the case of HDR irradiation, the percentage of DNA damage was 5 and 8% for irradiation at doses of 4 and 8 Gy, respectively. After LDR irradiation, it was 3 and 4% after irradiation in similar doses. The absence of a radiation arrest of mitoses after LDR irradiation is most likely associated with an insufficient level of DNA damage. In turn, the different values of the number of DNA breaks after irradiation in the same doses may be associated with the ongoing repair processes during LDR irradiation.

### 3.6. Phosphatidylserine Externalization

One of the commonly considered signs of regulated cell death is the loss of plasma membrane asymmetry manifested with the exposition of phosphatidylserine on its outer surface. This process is usually associated with apoptotic death, but in some cases, it can also be detected in necroptotic or pyroptotic cells [44].

The mechanism of cell death was studied by staining cells with AnnexinV-FITC (AnxV-FITC) and the nuclear dye propidium iodide (PI) using flow cytometry. AnxV is a protein that specifically binds to phosphatidylserine which becomes exposed on the outer leaflet of the plasma membrane at the early stages of apoptosis. PI is a stain that binds to double-stranded DNA and can enter the cells when the cell membrane loses its integrity. AnxV-FITC staining in the absence of PI staining corresponds to early apoptosis without compromising membrane integrity («PI-AnnV+»). «PI + AnxV-» and «PI + AnxV+» cells can be considered as dead cells with impaired cell membrane integrity. We must note that phosphatidylserine exposure itself is not the irreversible stage in cell death processes. It has been experimentally proven that «AnxV+» apoptotic cells are able to undergo anastasis, the process of recovery of the cell functionality after the initiation of cell death machinery. In this case, the cell counted as “death” will resurrect [45,46,47]. This point should be taken into account for the correct interpretation of the fate of the population of cells labeled as “early apoptotic”.

**Figure 5:**
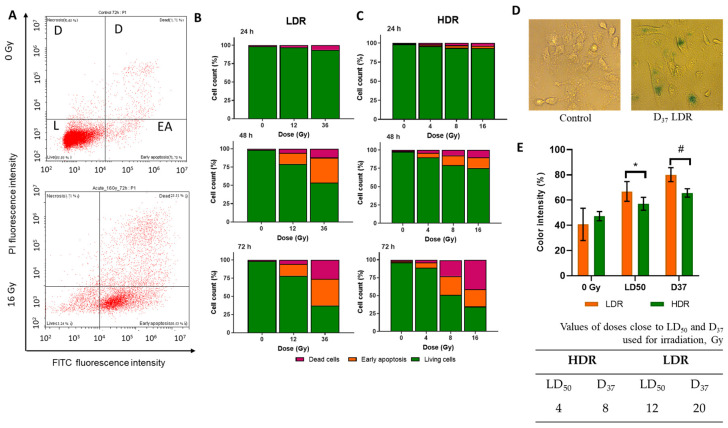
(**A**)—example of the distribution of cells into live («PI-AnnV-») (L), early apoptotic («PI-AnnV+») (EA), and dead («PI+») (D) in the non-irradiated cells and cells after 72 h after HDR irradiation; (**B**)—the distribution of the A431 cells into live («PI-AnnV-»), early apoptotic («PI-AnnV+»), and dead («PI+») 24, 48, and 72 h after LDR irradiation at various doses; (**C**)—the distribution of the A431 cells into live, early apoptotic, and dead 24, 48, and 72 h after HDR irradiation at various doses; (**D**)— representative images of cells for SA-β-galactosidase assay; (**E**)—the activation of β-galactosidase in A431 72 h after HDR and LDR irradiations at LD_50_ and D_37_. Data are represented as mean ± CV (*n* = 3), where “CV” is the coefficient of variation. “*” and “#” indicate significant differences in the color intensity between the non-irradiated and irradiated cells (ANOVA and Dunnett’s test, * *p* < 0.05; # *p* < 0.0001) Error bars represent the coefficient of variation (CV). The non-irradiated cells were used as control.

Irradiation in both modes of exposure causes an increase in the number of «PI+» and cells of «AnnV+» (Figure 5A). This effect is dose-dependent, i.e., in both modes, with increasing dose, the proportion of both «PI+» and cells «PI + AnnV-» increases (Figure 5B,C). The time dynamics of the distribution of cells across populations fall into the following patterns: 24 h after irradiation, a slight decrease in the proportion of «PI-AnnV-» is observed under both irradiation modes; 48 h after irradiation, the proportion of both cells «PI-AnnV+» and «PI+» increases significantly. Moreover, after LDR irradiation, the percentage of «PI-AnnV-» cells is significantly lower than after HDR irradiation at the corresponding doses. After LDR irradiation, the percentage of «PI-AnnV-» cells was 50–75% (Figure 5B). In the case of HDR irradiation, it was 75–85%. A duration of 72 h after irradiation, the percentage of «PI-AnnV-» cells decreased even more. In particular, after HDR irradiation, the percentage of «PI-AnnV-» cells was 60–80%, and after LDR irradiation, it was 60–75%. Additionally, after HDR irradiation, the percentage of «PI+» cells increased more than the percentage of cells «PI-AnnV+» (Figure 5C). At doses well above D37 (36Gy LDR and 16Gy HDR), we observe the same pattern in the ratio of «PI+» to «PI-AnnV+» cells 72 h after irradiation. Phosphatidylserine on the outer surface of the membrane is not an unambiguous criterion for imminent cell death. Cells «PI-AnnV+» have several options for the path of their fate. They can avoid death and return to their normal functioning, they can transform into anastatic cells (which we write about in point 12), or they can die and replenish the pool of dead cells [48,49,50].

Comparing the characteristics of the response dynamics for LDR and HDR irradiations, it is important to note that under LDR irradiation, there is a larger percentage of cells «PI-AnnV+» compared to «PI+» cells. Thus, after 48 h, the ratio of cells «PI-AnnV+» to «PI+» cells was 4 to 1 under LDR irradiation and 1 to 1 under HDR irradiation.

We have shown a statistically significant increase in the intensity of cell staining using SA-beta-galactosidase staining after LDR irradiation. This increase is dose-dependent. In the case of irradiation at a dose of LD_50_, the increase was 1.5 times. After irradiation at a dose of D37, it was two times. No statistically significant differences were observed after HDR irradiation (Figure 5D). Cellular senescence is one of the variants of consequences after radiation damage to the cell.

### 3.7. ROS Levels in Cells

**Figure 6:**
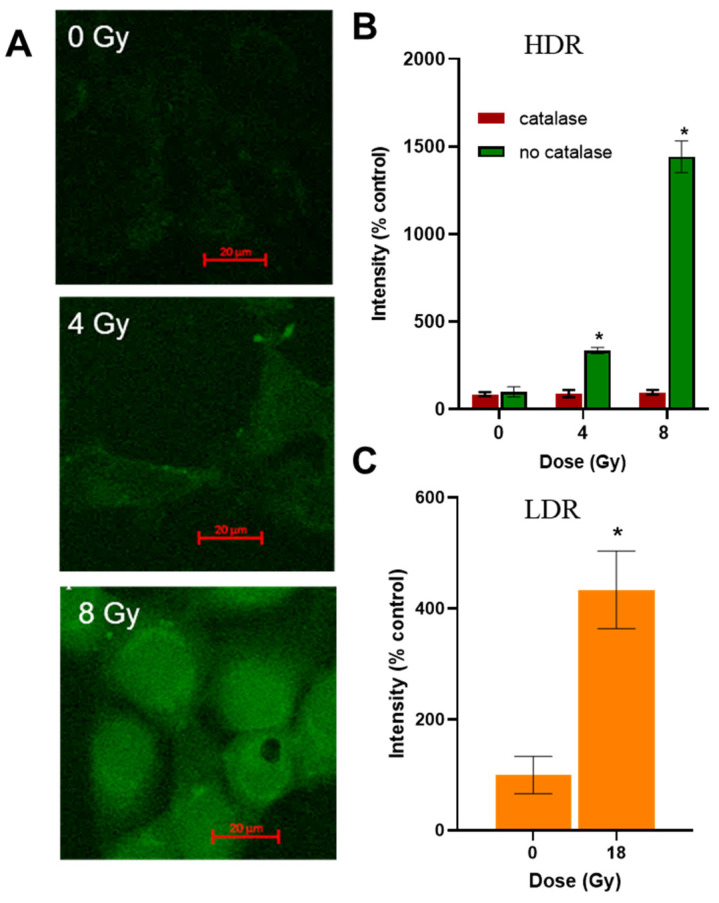
Assessment of hydrogen peroxide level in the A431 cells after HDR and LDR irradiations. (**A**)—images of the A431 cells treated with ROS-sensitive fluorescent probe DCFH_2_DA and HDR irradiation; (**B**)—hydrogen peroxide levels in the A431 cells 40 min after HDR irradiation at various doses; (**C**)—hydrogen peroxide level in the A431 cells 40 min after LDR irradiation at a dose of 18 Gy; data are represented as mean ± CV, where CV is the coefficient of variation; “*” indicates significant differences in the fluorescence intensity of the probe in the non-irradiated and irradiated cells (Wilcoxon’s test, * *p* < 0.0001).

The level of hydrogen peroxide in cells after irradiation was assessed using the fluorescent probe DCFH_2_DA. After HDR irradiation, an increase in the fluorescence intensity of the probe was observed 40 min after the end of irradiation (Figure 6A). When the cells were preincubated with catalase, the fluorescence intensity of the probe did not increase (Figure 6B). After LDR irradiation at a dose close to LD_50_, the fluorescence intensity of the probe increased four times (Figure 6C). When irradiated in the HDR mode at a dose of 8 Gy (D_37_), the fluorescence intensity of the probe increased 15 times (Figure 6B). To confirm the formation of ROS, we present data in Figure S2 on the generation of hydrogen peroxide after irradiation.

### 3.8. Giant Cells

**Figure 7:**
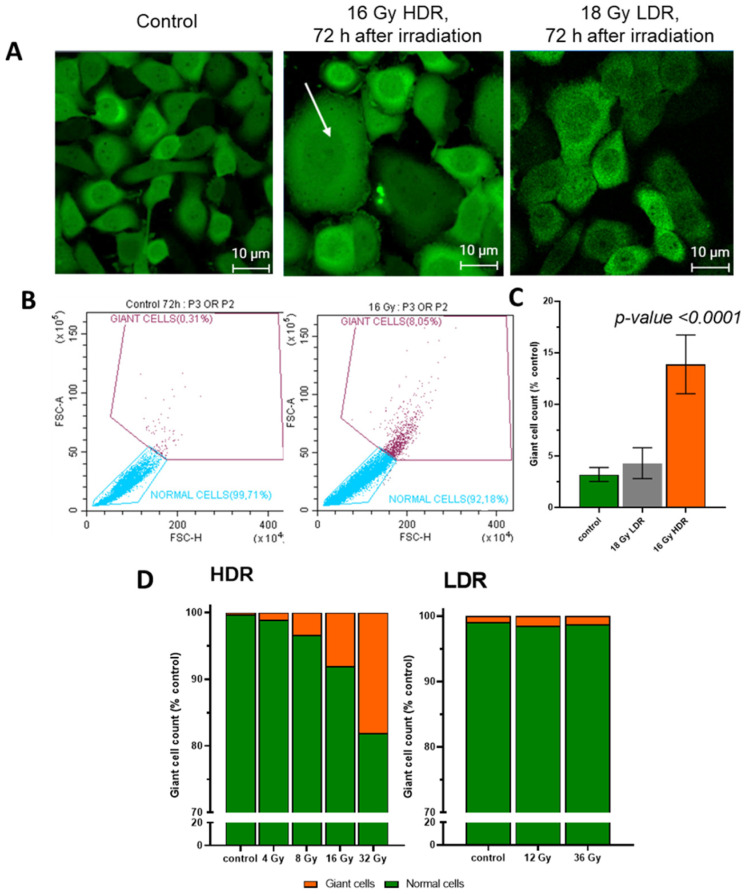
(**A**)—images of A431 72 h after HDR and LDR irradiations with the formation of “giant cells”. The arrow indicates "giant cells"; (**B**)—the relative number of “giant cells” 72 h after LDR and HDR irradiations; (**C**)—distribution of cells into normal cells and “giant cells” in the non-irradiated and HDR-irradiated culture; (**D**)—the distribution of the A431 cells into normal cells and “giant cells” 72 h after irradiation at various dose rates. Error bars represent the coefficient of variation (CV).

After HDR irradiation, morphological changes in the cells were observed, namely the formation of giant cells. The area of giant cells exceeded the area of average cells by 3–7 times (Figure 7A,C). When irradiated in the HDR mode at a dose of 16 Gy, the number of giant cells in the irradiated culture exceeded the number in the non-irradiated culture by five times (Figure 7B). After LDR irradiation even at high doses, we did not observe a significant increase in the number of giant cells (Figure 7B,D). The emergence of giant cells may be associated with mitotic catastrophe or errors in the resolution of the radiation arrest of mitoses. According to our assumptions, giant cells make an important contribution to delayed cell death after irradiation. It is worth noting that we observe an increase in cell size after LDR irradiation by 1.5–2 times. We attribute this to the senescence processes that take place after LDR irradiation [38]. We do not call them giant cells, as they are much smaller in size than those observed after HDR irradiation.

## 4. Discussion

The effect of ionizing radiation on cells significantly depends on the particle fluence, which leads to the so-called dose-rate effects [51], e.g., different cell viability after irradiation at different dose rates [52,53]. Dose-rate effects manifest in varying degrees in different cells, particularly in normal and tumor cells. Tumor cells differ from normal cells with a higher proliferation rate, absence of replication limit, glycolysis-biased metabolism, and altered gene expression of key cell signaling pathways, including pathways that trigger apoptosis [54,55]. Generally, normal cells exhibit higher radioresistance compared to tumor cells due to the correct operation of additional cell survival pathways [56,57]. In terms of dose-rate effects, normal cells are less sensitive to LDR irradiation than to HDR irradiation [58]. This makes the use of LDR irradiation promising for reducing radiation exposure and possible mutagenic activity on normal cells in the tumor node [59]. However, it should be noted that dose-rate effects are not entirely straightforward, e.g., there are variations in dose-rate susceptibility among different tumors [58]. So, to provide an objective view of dose-rate effects in different tumors, their underlying mechanisms should be unraveled.

To compare the mechanisms of the response of tumor cells to low- and high-dose-rate irradiation, we have selected dose rates corresponding to those used in cancer radiotherapy, namely to low-dose-rate brachytherapy (0.4–5 Gy/h, LDR) [7] and to electron beam therapy (≈600 Gy/h, HDR) [60], and applied these modes of irradiation to human epidermoid carcinoma A431 cells. We demonstrated significantly more pronounced radiosensitivity of A431 cells to HDR irradiation compared to LDR irradiation. Also, we registered a more pronounced decrease in cell growth rate after HDR irradiation compared to LDR irradiation at similar doses (Figure 3). This difference is associated with a radiation-induced halt of mitosis, i.e., cell cycle arrest at the G2/M phase, occurring in cells exposed to HDR irradiation, while in cells exposed to LDR irradiation the cell cycle is not altered (Figure 4B,C).

The radiation-induced halt of mitosis is a typical mechanism of cell radioprotection which is triggered by exceeding a certain threshold in the degree of DNA damage [61]. Cells can overcome the radiation halt of mitosis if critical DNA errors are successfully repaired within several doubling cycles, otherwise, apoptosis or autophagy are triggered [62,63,64]. We showed a significantly higher degree of DNA damage under HDR irradiation compared to LDR irradiation (Figure 4D). Immediate and prolonged (at higher doses) cell cycle arrest in cells exposed to HDR can be explained by severe DNA damage occurring by the combined effect of direct DNA damage by ionizing radiation and oxidative stress triggered by ROS [65]. Oxidative stress arises due to damage to the mitochondria, which are organelles characterized by an increased level of ROS [66]. Their damage leads to the disruption of the electron transport chain and the emergence of a secondary wave of ROS formation in the cell [67,68,69]. Oxidative stress such a long time after irradiation may indicate secondary ROS generated as a result of mitochondrial damage [70].

ROS may also contribute to cell cycle arrest acting as secondary messengers, participating in intracellular and intercellular signaling [71,72,73,74]. In the case of intracellular signaling, they participate in the pathways of cyclin-dependent kinases (CDKs). Our work demonstrates that cell cycle arrest in the cells exposed to HDR irradiation occurs in the G2/M phase. During mitosis, the transition from the G2 phase to mitosis is controlled by the cyclin B1/CDK1 complex [75,76,77]. It has been reported that this cyclin B1/CDK1 complex is associated with mitochondrial activity as well as with the activation of cell radioprotection mechanisms [78,79,80]. In the case of intercellular signaling, ROS participate in the so-called bystander effect, i.e., the death of the cells which were not directly damaged by irradiation, but were immersed in a common medium with irradiated cells [81,82]. This effect can also contribute to delayed cell death after HDR irradiation; however, the question if it is involved in the induction of cell cycle arrest should be clarified.

An interesting manifestation, proving that the halt of mitosis is the main defensive mechanism of the response of A431 cells to HDR irradiation, is the emergence of “giant cells” (Figure 7). This is associated with the inability to repair DNA damage and to complete mitosis or with the fusion of several cells with each other [83]. In terms of post-irradiation tumor development, the emergence of “giant cells” has two contradicting aspects. On the one hand, “giant cells” reduce the tumor growth rate due to the impossibility of their division and death. However, they are reported to participate in the process of metastasis [84,85] and to influence neighboring cells, including the differentiation of stem cells [86]. Thus, the formation of “giant cells” may be a marker of a tumor node acquiring potential aggressive behavior and a factor in tumor relapse after therapy [87]. Moreover, it is known that DNA repair can occur already during mitosis, which can lead to subsequent genetic instability, cell death, or mutations [88]. Thus, we can talk about the potential danger of HDR irradiation over LDR irradiation due to the formation of “giant cells”. Further study of the mechanisms of the formation of these cells, as well as their characteristics, will allow us to better understand the likely outcome of the tumor behavior after HDR irradiation. “Giant cells” have a clear advantage over cancer therapy and contribute to tumor resistance, but they can also be a biomarker for assessing the effectiveness of therapy and adjusting treatment strategy [89]. It is known that senescence can lead to the formation of “giant cells”. In senescent cells, morphology, organelle function, chromatin organization, gene expression, and metabolism change lead to the acquisition of a pro-inflammatory phenotype known as the senescence-associated secretory phenotype [90,91]. In turn, we observed an increase in cell size after LDR irradiation, most likely associated with senescence, but it was insignificant (1.5–2 times) compared to the size of “giant cells” after HDR irradiation (3–7 times). It is worth conducting additional studies on the manifestation of radioresistance and the fate of such “giant cells” after both modes of irradiation.

The registered levels of oxidative stress 40 min in cells after LDR and HDR irradiations (Figure 6) indicate that mechanisms for the initial development of radiation damage to cells induced by different dose rates are similar. However, cells exposed to LDR irradiation do not experience a halt of mitosis even when exposed to much higher doses compared to HDR irradiation (Figure 3). Regarding the cell, fate is determined by the competition of cell damage and the functioning of repair systems, and cell cycle arrest can occur after exceeding a threshold in DNA damage; we assume that this threshold is not exceeded in the case of LDR irradiation. The insufficient DNA damage also explains a milder decrease in culture growth rate after LDR irradiation (Table 2), because the reduction in the number of cells was most likely due to the elimination only of cells that died during the first day after irradiation. Taking into account that the contribution of ROS to the damage of DNA should be similar under either mode of irradiation (Figure 6), we naturally conclude that the main reason for insufficient DNA damage under LDR irradiation is the insufficient direct irradiation-induced damage or the adaptation processes of the cell. It should be noted that our data on ROS generation in cells do not indicate the same mechanisms of oxidative stress occurrence and the development of a response to it after HDR and LDR irradiations. Since LDR irradiation is carried out within 24 h, many adaptive mechanisms are definitely involved [92,93,94]. In addition to the classical mechanisms of combating oxidative stress, physiological processes in cells will also have time to change during such a long period of exposure. Thus, in our opinion, the role of ROS generation in the development of cell response after HDR and LDR irradiations requires additional in-depth study. In addition to adaptation mechanisms, it is necessary to take into account that under LDR irradiation the particle fluence is low, so the comparable-to-HDR-irradiation amount of energy is transferred to a certain microvolume (target compartment) of the cell in a longer time. So, the cell has time to repair the damage before the same compartment receives energy next time [19,27]. This repair can be executed by different pathways of DNA double-strand break repair, e.g., the pathway based on homologous recombination, non-homologous end joining, and alternative end joining [95]. The exact pathways by which the cell selects the repair strategy are unknown. It is believed that repair mechanisms may be specific for different types of damage [25]. It is worth additionally studying the dynamics of the occurrence of DNA damage in the process of LDR irradiation, as well as the dynamics of the repair of these DNA damages at various intervals after irradiation in both modes of exposure.

Among the radioprotective mechanisms alternative to the radiation-induced halt of mitosis, there are mechanisms of cellular senescence [96,97,98]. We demonstrate that after LDR irradiation, the senescence phenotype is actively formed (Figure 5D). The underlying mechanisms here can be (i) p53-dependent senescence based on the activation of AMP-dependent protein kinase, which is associated with mitochondrial dysfunction; (ii) the activation of proteins of the RAS family, leading to glycolysis-biased metabolic switch and the additional activation of the hypoxia factors HIF1α [99,100]. Most likely, the first way should not be considered in relation to our cell culture in connection with the inactivated p53. We should note that the activation of cellular senescence in tumor cells can lead to more aggressive behavior of the tumor, e.g., the activation of epithelial-to-mesenchymal transition, metastasis, neoangiogenesis, and modification of the microenvironment [101,102]. A precise understanding of the mechanisms of senescence in the case of LDR irradiation is required to reduce the negative impact of cellular senescence in the long-term post-irradiation perspective.

Interestingly, LDR irradiation also has a positive dimension in the long-term post-irradiation perspective. We demonstrate that LDR irradiation induces regulated cell death which is most likely apoptotic in nature according to the presence of phosphatidylserine on the outer side of the membrane. The externalization of phosphatidylserine is necessary for its presentation to macrophages and subsequent cell death [44]. However, it should be noted that the release of phosphatidylserine does not mean the final start of apoptosis. The result may be the formation of anastatic cells [45,50]. Membrane symmetry violation and the externalization of phosphatidylserine are provided by an enzyme from the flippase group—scramblase. The important participation of scramblase in the processes regulating apoptosis and autophagy is known [44,103]. Apoptosis is an immunogenic and tolerogenic type of cell death [104]. The advantage of immunogenic–tolerogenic cell death is the activation of the adaptive immune response which reinforces anti-tumor immunity [105,106]; hence, it is a popular trend in modern science to search for immunogenic and tolerogenic treatment options. Radiotherapy in certain modes of exposure is one of the types of treatment that causes tolerogenic cell death [107,108]. Further study of the molecular mechanisms of cell response to different types of irradiations will potentially reveal ways to control the production of damage-associated molecular patterns and the activation of the immune system. Thus, the study of how tumor cells die remains an unsolved task in radiobiology, and it is more advantageous to trigger immunogenic and tolerogenic cell death by the mode of exposure.

We have shown that the main mechanism underlying the effects of LDR and HDR irradiations is the cell cycle arrest after HDR irradiation and the absence of such arrest after LDR. This difference is determined by the higher degree of DNA damage in HDR irradiation due to a more pronounced contribution of rapid radiation damage, as well as the late initiation of adaptation mechanisms. More subtle but long-term perspective critical points distinguishing the effects of LDR irradiation are the activation of senescence phenotype and possible induction of apoptosis. Due to their contradictory potential impact on tumor development, further investigations are needed to improve the existing treatment protocols. One promising option may be the combination of two irradiation options. Fractionated sequential HDR irradiation may allow the effects of increasing the radiosensitivity of tumor cells for the LDR treatment option or transition to ultrahypofractionated irradiation [109].

## Figures and Tables

**Table 1:** Characteristics of beta-emission sealed sources to achieve LDRs.

Radiation Activity, MBq	Modification	Dose Rate, Gy/h
1	shielding with films	0.25
1	-	0.5
3	shielding with films	0.75
1	combining two sources	1
3	-	1.5
3	combining two sources	3

**Table 2:** A431 growth rate 72 h after HDR and LDR irradiations at doses in the neighborhood of LD_50_ and D_37_.

Control	HDR		LDR	
**–**	**LD_50_ (MTT)**	**>D_37_ (MTT)**	**LD_50_ (MTT)**	**>D_37_ (MTT)**
1.2	0.7	0.2	1.0	0.85

After HDR irradiation, the slowdown in cell growth in culture was more pronounced compared to the LDR irradiation.

## Data Availability

The original contributions presented in the study are included in the article; further inquiries can be directed to the corresponding author.

## Supplementary Materials

The following supporting information can be downloaded at https://www.mdpi.com/article/10.3390/cimb46120828/s1, Figure S1: A representative image of a giant cell after HDR irradiation, 16 Gy and 72 h; Figure S2: Dynamics of hydrogen peroxide concentration in the A431 cells stably transfected by a fluorescent sensor to HyPer peroxide after LDR irradiation, dose 0.125 Gy.
