# Supplementary figures and images for: The Dose Rate of Corpuscular Ionizing Radiation Strongly Influences the Severity of DNA Damage, Cell Cycle Progression and Cellular Senescence in Human Epidermoid Carcinoma Cells
Source: Curr Issues Mol Biol. 2024 Dec 6;46(12):13860–80. doi: 10.3390/cimb46120828 (PMC11726848; doi:10.3390/cimb46120828)

Supplementary

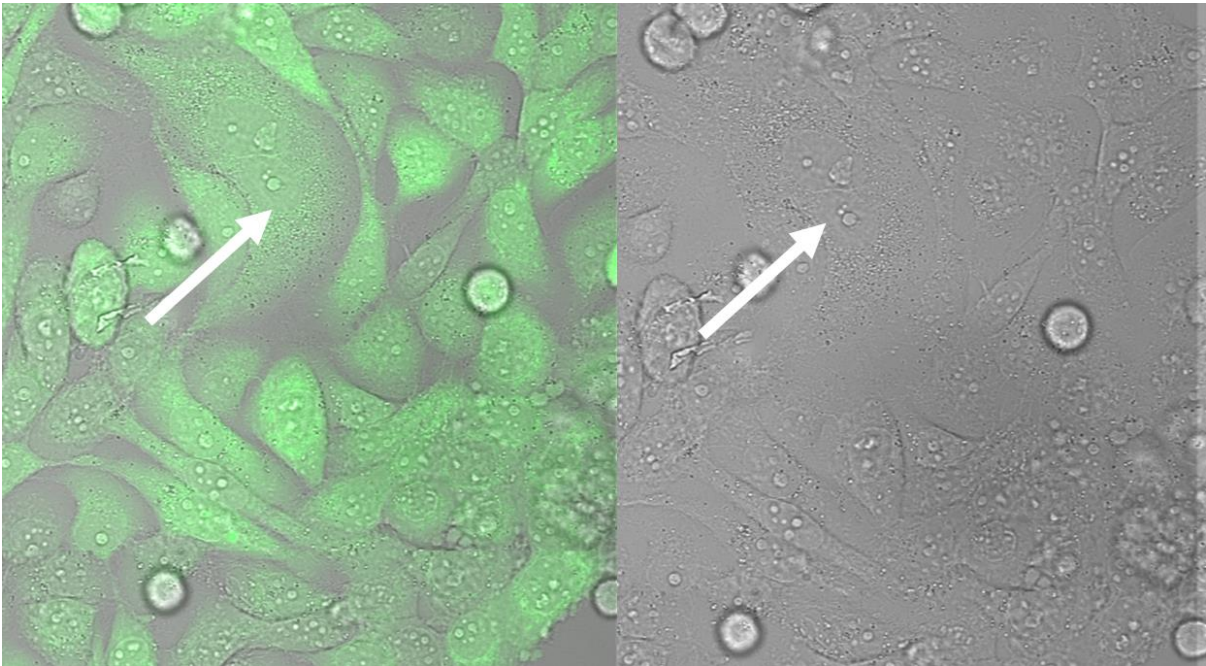

Suppl Figure S1.

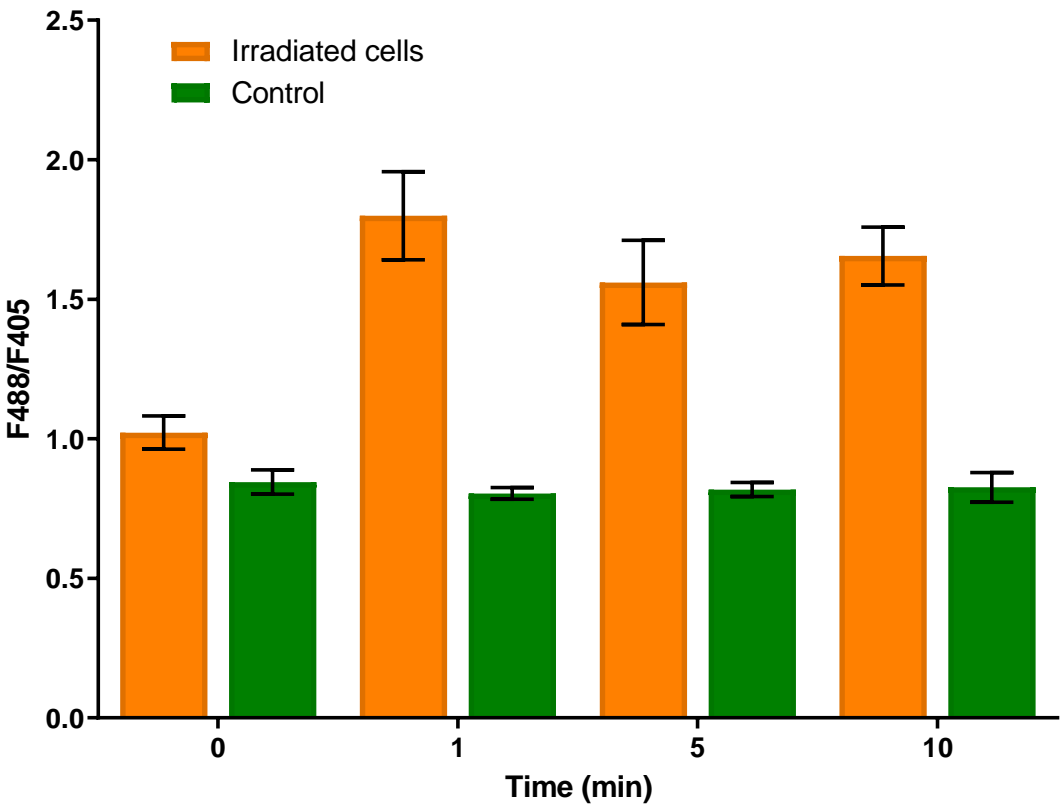

Suppl Figure S2.

Supplement: Supplementary file 1 [file cimb-46-00828-s001.zip › cimb-3305746-supplementary.pdf]
